# Experimental systems are essential for strengthening ecological modeling of microbiomes beyond observational data

**DOI:** 10.1128/msystems.01745-24

**Published:** 2025-07-21

**Authors:** Ezgi Özkurt

**Affiliations:** 1 Quadram Institute Bioscience, Food, Microbiome & Health Department, Norwich, United Kingdom; Wageningen University, Wageningen, the Netherlands

**Keywords:** microbiome assembly, community ecology, ecological models, microbiome stochasticity

## Abstract

Disentangling the ecological mechanisms shaping the assembly of complex communities with thousands of interacting species remains a significant challenge. Ecological models derived from observational data are valuable tools for describing community states and generating hypotheses. Integrating these models with experimental approaches is crucial for addressing the challenges of uncovering the complex mechanisms and dynamics underlying microbiome assembly. Strategic experimental designs can complement observational data, improving inference accuracy and advancing efforts to restore microbiota and enhance their therapeutic potential. Building on insights from previous studies, this paper organizes core concepts into four main themes where controlled, trackable experiments are particularly effective in advancing our understanding of microbiome assembly rules and mechanisms: resolving the role of (i) ecological drift and (ii) priority effects in microbiome assembly, (iii) subspecies-level microbial dynamics, and (iv) controlled replication of community assembly dynamics.

## PERSPECTIVE

Applying community ecology principles to observational microbiome data presents substantial challenges, especially given the complexity of microbial interactions. Even in controlled experiments with only two microbial strains, interactions can be unexpectedly intricate (1, 2), underscoring the difficulty of inferring detailed community dynamics from observational data alone. Additionally, microbiome composition varies widely among individual hosts (3); for instance, only a small fraction of microbial species are consistently shared across human microbiomes (4, 5). This variability arises from factors such as diet (6), genetics (7), geography (8), gender (9), and medication (10), though random events (11) also play a substantial yet often underexplored role. Disentangling the deterministic and stochastic processes driving this variability requires comprehensive metadata collection. However, confounding variables often complicate analysis (12, 13), and it is impossible to fully capture all host-associated factors or confounders without controlled conditions. Consequently, observational (top-down) studies alone offer limited insight into the causes and consequences of microbiome variation. Integrating observational data with controlled (bottom-up) experiments under identical conditions holds great promise for revealing baseline stochasticity and the deterministic factors shaping microbial communities. Understanding these ecological processes in-depth is essential to the success of therapeutic microbiome interventions like fecal microbiota transplantation (FMT) and personalized healthcare, where context-dependent factors and historical contingencies contribute to variable outcomes (14–16). Leveraging these insights enables systematic examination of assembly dynamics, uncovering the ecological principles that govern these complex and variable communities.

**Fig 1:**
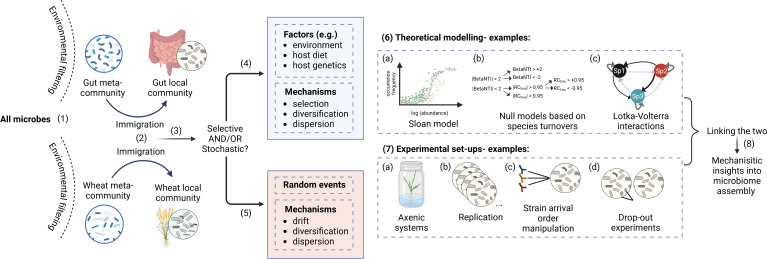
Mechanisms of microbiome assembly and approaches to uncover the ecological forces driving it. Only a subset of microbes from the environment can colonize different hosts due to environmental filtering (1). From these filtered microbes (i.e., the metacommunity or regional source for each host environment, e.g., gut or wheat microbiomes), only a smaller subset is able to colonize each individual host (i.e., local community) (2). This immigration of microbes from the metacommunity to the local community can be shaped by deterministic or stochastic processes or a combination of both (3). Both types of processes are driven by different ecological factors and mechanisms (4, 5). Theoretical ecological models (6) are often used to infer community assembly dynamics, including (a) neutral models (e.g., Sloan model), which assume all taxa have equal fitness and that community structure is shaped mainly by random dispersal and drift; (b) null models based on phylogenetic or taxonomic turnover (e.g., beta nearest taxon index [βNTI], Raup-Crick metric based on Bray-Curtis dissimilarity [RC-Bray]), which assess whether observed community patterns deviate from random expectations; (c) Lotka-Volterra models, which use time-series data to estimate species interactions and infer community stability or dynamics. Various experimental setups (7) provide insights into the role of these processes, such as (a) using axenic hosts to monitor microbial colonization dynamics, (b) replicating microbiome assembly under controlled conditions, (c) manipulating colonization order, and (d) altering microbiome composition to assess the roles of individual microbes in the community. Integrating experimental setups with ecological models (8) creates a complementary approach, enabling a transition from descriptive studies to mechanistic insights into microbiome assembly and dynamics. Created in https://BioRender.com.

In this perspective, I address the limitations of ecological models derived solely from observational data in understanding the mechanisms driving microbiome assembly and explore how experimental approaches can help address these challenges (Fig. 1).

### The entangled complexity of ecological drift and other ecological forces

Ecological drift, or demographic stochasticity, refers to random fluctuations in population size that significantly affect community assembly (Fig. 1). It significantly affects community structure, particularly in rare species that recently immigrated or remained at low abundance after disturbances like antibiotic treatment (17). However, certain conditions, such as selection pressures favoring “rescue” of these rare species or external replenishment, may allow them to persist within the system. In low-biomass microbiomes, such as those on human skin or plant seeds, effects of drift are amplified, particularly during early microbial colonization phases (18). For instance, neonatal microbiome colonization, in which microbial populations are sparse (18), may be more susceptible to drift than to selective processes.

In community ecology, drift is often inferred from single population snapshots, making it hard to distinguish from dispersal or weak selection (19). Observational data alone are insufficient, and controlled experiments are needed to quantify drift accurately.

Fodelianakis et al. (19) isolated ecological drift in simplified bacterial communities using flow cytometry and mathematical models (19). Their findings showed drift’s influence increased under high selection pressure and low dispersal, highlighting its role in natural community variability.

Overall, these findings lay the groundwork for future studies investigating ecological drift in more complex microbial systems, particularly when methodological limitations of ecological models reliant solely on observational data are addressed.

### Resolving the role of priority effects in microbiome assembly

Priority effects—a “time-explicit”' form of dispersal that can act as either a stochastic or deterministic factor depending on the mechanism (Fig. 1)—occur when the arrival order of species influences microbiome assembly, with significant impacts during primary ecological succession (20–24). Priority effects arise when early colonizers pre-empt or modify niches, either inhibiting (“niche pre-emption”) or facilitating (“niche facilitation”) the establishment of later species (25).

Numerous studies have examined the role of priority effects in microbiome assembly in different hosts (23, 26–31). Our understanding of priority effects largely stems from two-species Lotka-Volterra (L-V) models, which are limited in addressing multispecies microbiomes (32, 33).

Unlike mechanistic models that explicitly include interaction mediators, L-V models consider only net fitness effects, limiting their applicability to microbiomes where interactions are often chemically mediated. They also fall short in multispecies systems, as they cannot capture indirect interactions, thereby violating the additivity assumption (33). While observational data can generate useful theoretical predictions based on L-V models (34), these must be validated and further explored through experimental approaches. Manipulating factors such as species arrival order, frequency, phylogenetic relatedness, and host selectivity can offer deeper insights into these dynamics.

Experimental studies have shown that early colonizers can create alternative community states, as seen in a porcine model where stochastic *Clostridia* colonization shaped microbiome assembly (35). In mouse gut models, niche overlap and phylogenetic relatedness amplified priority effects, with early-arriving species pre-empting niches for others of the same species while allowing colonization by different species (36, 37). These studies have provided in-depth insights by documenting species arrival histories under controlled conditions. Integrating theoretical predictions with these experimental setups is essential for unraveling the complexity of priority effects in microbiomes and understanding how they drive the emergence of alternative stable states within hosts.

Agent-based models (ABMs) are commonly used to model microbe-microbe interactions in microbial communities (38, 39), for example, in synthetic gut microbiome communities (40, 41). In co-culture setups, growth kinetics and metabolite production profiles of the interacting microbes can be modeled to gain mechanistic insights into the dynamics of priority effects during microbiome colonization. Modeling based on ordinary differential equations (ODEs) is particularly useful for this purpose (42). However, unknown parameters can reduce the accuracy of ODE-based models, making it essential to estimate them using experimental data and validate the model with independent data sets.

Hybrid models, such as those integrating ABMs and metabolic networks (40) or the ones coupling ODE models with LV equations and ABMs—which often incorporate sequence data—can provide a deeper understanding of microbe-microbe interactions within microbial ecosystems (43). These models can also clarify the role of priority effects in microbiome assembly when the findings are further explored on co-culture experiments and used to model the growth kinetics and metabolite production profiles of the interacting microbes.

### Subspecies level as an important ecological unit, but hard to study with existing ecological models based solely on observational data

Microbial community dynamics occur below the species level, where species encompass numerous genetic variants (44, 45). For example, while mother-infant microbiomes converge at the species level early in life, they diverge at the strain level over time (46). Similarly, while multiple *Cutibacterium acnes* lineages inhabit human skin, individual pores often host only a single lineage, underscoring spatial strain-level variation (47). Strains are thus critical ecological units, but whether they follow macroecological patterns seen at the species level or exhibit unique assembly dynamics remains unclear (48).

Studying subspecies-level assembly using purely observational data is challenging due to sequencing limitations. Short-read amplicon sequencing typically resolves only genus or species levels at best (49, 50). Full-length amplicon sequencing offers higher resolution but struggles to resolve strains due to slower marker gene evolution (51). Metagenomic sequencing enables strain-level insights (20, 32); however, this can be challenging, as short-read sequencing struggles with repetitive regions, and long-read sequencing suffers from high error rates (52). Some mathematical models, for example, strain-specific genome-scale metabolic models (GEMs), built from the core and pan-genome of bacterial species (53, 54), can aid in understanding subspecies-level dynamics in microbiome sequence data sets. However, these models still face limitations due to current sequencing technologies, which hinder accurate subspecies-level analysis from observational data alone.

Understanding the roles of individual strains within microbial communities presents a complex challenge—one that experimental systems are well-suited to address. For instance, research using germ-free mice colonized with a diverse synthetic community systematically examined the impact of individual strain loss (55). Strikingly, removing just two species led to complete loss of a metabolic pathway. While reductionist, this approach provides valuable mechanistic insights into subspecies interactions that complement observational studies.

The need to study strain-level dynamics is further emphasized by the substantial variation observed in the human gut microbiome, where less than 5% of strains are shared between individuals (45). Recent studies highlight the importance of this variability (56–58). Strain abundances in the human gut are predicted to follow a stochastic logistic model, fluctuating around a fixed carrying capacity (48). However, linking these patterns to causal mechanisms requires experimental setups that bridge observational findings with mechanistic insights. Notably, certain strains, such as strains from *Faecalibacterium prausnitzii*, deviate from predicted patterns, with rapid strain turnover likely driven by inter-strain competition or other factors (48)—a hypothesis that experimental models should further investigate.

Importantly, studies also show that only a few strains colonize the human gut at any given time, with a single strain typically dominating each species (45). This “oligocolonization” is believed to be governed by ecological constraints (45, 48, 59), though the mechanisms limiting colonization to a few strains remain unknown—a question that experimental setups informed by ecological models could help resolve.

In summary, experimental systems are essential for uncovering strain-specific ecological processes that observational data alone cannot resolve. Bridging theory with experiments will enhance our understanding of microbial assembly at higher genomic resolution.

### The importance of controlled replication in understanding stochasticity in microbiome assembly

A major challenge in observational microbiome studies is achieving sufficient replication to identify consistent ecological dynamics. This limitation introduces noise, making it difficult to disentangle stochasticity from selection (60). Experimental systems provide controlled conditions with high replication, enabling deeper insights into microbial assembly and its repeatability. Laboratory models, such as axenic or gnotobiotic systems, are particularly effective for studying microbial assembly dynamics *in vivo* with replication under controlled conditions that preserve the host-associated environment as the natural context for microbial assembly. Unlike liquid cultures, these systems incorporate crucial host factors—such as immunity—that significantly influence microbiome dynamics.

Maignien et al. (61) used replicate *Arabidopsis thaliana* plants grown in identical greenhouse conditions to track phyllosphere microbiome dynamics (61). Their results showed that replicate communities consistently converged toward a similar composition, highlighting the role of selective processes in shaping microbial assembly.

Özkurt et al. exploited the power of seeds from the same plants as biological replicates to study the assembly of microbial communities transmitted from seeds to newly developing wheat plants (62). They found that seeds from the same spike shared similar microbiomes, while seedling microbiomes showed greater variability. This demonstrated how selective processes shape the vertically transmitted microbes in wheat, while further assembly during seedling development is influenced by stochastic factors (62).

Animal model studies also illustrate the power of replication under controlled conditions. Burns et al. (63) investigated the assembly of the zebrafish gut microbiome throughout host development by studying a population derived from a single mating pair and raised under identical conditions (63). This setup minimized variability, providing clear insights into microbial colonization dynamics.

These studies emphasize the importance of replication in controlled experimental systems for disentangling stochastic and deterministic processes in microbiome assembly. While stochastic processes can result in microbial communities with distinct or overlapping metabolic functions, understanding these functional outcomes requires more than taxonomic profiling. For example, GEMs (64) provide a powerful framework for predicting metabolic potential, functional redundancy, and cross-feeding interactions (65, 66). These models are most informative when applied to microbiomes assembled under standardized, replicated conditions, where metabolic potentials of alternative microbiome states can be systematically explored, reinforcing that standardized, repeatable conditions are essential to identify the ecological mechanisms shaping microbial communities.

### Concluding remarks

Future advancements in microbiome research technologies, such as ultra-resolution metagenomics and precise absolute quantification, will allow higher-resolution analyses of microbiome data sets, offering greater ecological and mechanistic insights. Nevertheless, the challenges highlighted here will persist, even with ideal observational data sets.

In this perspective, I highlight the importance of integrating controlled, trackable experiments with ecological models to address the limitations of ecological modeling relied on solely observational data. While excellent studies already leverage this synergy, further research is essential to overcome these challenges and enhance the successful bioapplication of microbiomes in therapeutic interventions by deepening our understanding of microbiome assembly and variability. For instance, optimizing seed inoculation with beneficial microbes for biocontrol in crop microbiomes—aimed at enhancing plant adaptation, yield, and resilience against pathogens—or improving the success of FMTs in restoring gut health can greatly benefit from the mechanistic insights that the integrated approaches offer.
